# Being recovered: a qualitative study of parents’ experience of their child’s recovery up to a year after a displaced distal radius fracture

**DOI:** 10.1302/2633-1462.55.BJO-2024-0024

**Published:** 2024-05-21

**Authors:** Emma E. Phelps, Elizabeth Tutton, Matthew L. Costa, Juul Achten, Phoebe Gibson, Amy Moscrop, Daniel C. Perry

**Affiliations:** 1 Nuffield Department of Orthopaedics, Rheumatology and Musculoskeletal Sciences, Kadoorie, Oxford Trauma and Emergency Care, Oxford University, Oxford, UK; 2 Oxford University Hospitals NHS Foundation Trust, John Radcliffe Hospital, Oxford, UK; 3 Alder Hey Children’s NHS Foundation Trust, Liverpool, UK; 4 Department of Child Health, University of Liverpool, Alder Hey Hospital, Liverpool, UK

**Keywords:** Trauma, Paediatrics, Qualitative research, Interviews, Displaced distal radius fracture, distal radius fractures, wrist, surgical casting, Randomized Controlled Trials, clinicians, internal fixation, wrist fracture, Kirschner (K)-wires, Fracture Fixation, bone remodelling

## Abstract

**Aims:**

The aim of this study was to explore parents’ experience of their child’s recovery, and their thoughts about their decision to enrol their child in a randomized controlled trial (RCT) of surgery versus non-surgical casting for a displaced distal radius fracture.

**Methods:**

A total of 20 parents of children from 13 hospitals participating in the RCT took part in an interview five to 11 months after injury. Interviews were informed by phenomenology and analyzed using thematic analysis.

**Results:**

Analysis of the findings identified the theme “being recovered”, which conveyed: 1) parents’ acceptance and belief that their child received the best treatment for them; 2) their memory of the psychological impact of the injury for their child; and 3) their pride in how their child coped with their cast and returned to activities. The process of recovery was underpinned by three elements of experience: accepting the treatment, supporting their child through challenges during recovery, and appreciating their child’s resilience. These findings extend our framework that highlights parents’ desire to protect their child during early recovery from injury, by making the right decision, worrying about recovery, and comforting their child.

**Conclusion:**

By one year after injury, parents in both treatment groups considered their child “recovered”. They had overcome early concerns about healing, the appearance of the wrist, and coping after cast removal. Greater educational support for families during recovery would enable parents and their child to cope with the uncertainty of recovery, particularly addressing the loss of confidence, worry about reinjury, and the appearance of their wrist.

Cite this article: *Bone Jt Open* 2024;5(5):426–434.

## Introduction

The Children’s Radius Acute Fracture Fixation Trial (CRAFFT) is a multicentre randomized trial of “surgical reduction” (which involved anatomical reduction under general anaesthetic (GA)/sedation with or without fixation with metalwork) versus non-surgical casting (which involved approximate alignment and plaster immobilization without GA/sedation) for displaced distal radius fractures.^1^ Semi-structured interviews exploring parents’ experience of the CRAFFT study and their child’s early recovery, up to three months after injury, highlighted the parents’ desire to “be a good parent and protect their child.”^2^ Parents endeavoured to make the right decisions about treatment and felt responsible for the decision to include their child in a RCT. The decision to have surgery or not was challenging, and some parents questioned whether they had made the right decision. Uncertainty about bone remodelling, deformity, and the substantial difference between the two treatments contributed to parents’ worry. However, parents had ongoing concerns about the appearance of their child’s wrist in both intervention groups.^2^

There is limited evidence regarding parents’ experience of their child’s recovery from fractures and how the experience changes over time. This study explores parent experience later in recovery at up to 11 months post-injury.

## Methods

The CRAFFT study is registered with the International Standard Randomized Controlled Trials Number Registry (ISRCTN10931294: 27 February 2020). The Black Country Research Ethics Committee approved the CRAFFT study, and this embedded qualitative study (REC reference 20/WM/0054).

A purposive sample of 20 parents (18 mothers, two fathers, one parent per child) from 13 NHS hospitals across the UK who were invited to take part in the CRAFFT study participated in a telephone interview. A total of 19 families had been randomized within the CRAFFT study; one had declined participation in the study, six children received surgical reduction, and 14 children received non-surgical casting. Ten children (four who had surgical reduction and six had non-surgical casting) had a completely offended fracture (the most severe fracture type). All children who were randomized received their allocated treatment. Children were aged between four and ten years (mean age 7.7 years (standard deviation (SD) 1.73)), with 12 males and eight females. Due to the researcher’s (EEP) maternity leave, interviews took place during two separate time periods, from October to December 2021, and January to July 2023. The children were all between five and 11 months post-injury (mean 8 months (SD 1.81)).

Families were informed of the qualitative study as part of the initial consent process for the CRAFFT study. Parents who were interested in being approached to take part in an interview provided electronic consent to be contacted. They were emailed an information sheet about the qualitative study and given the opportunity to ask questions. Parents who agreed to take part underwent a separate informed consent discussion, with verbal informed consent recorded and witnessed by an administrator who had undertaken research integrity training referred to as good clinical practice (GCP).

The interviews drew upon Heideggerian phenomenology and notions of Dasein (being or presence).^3^ This methodology has proved useful in studies of injury in adults, particularly where there is limited evidence of patient experience,^4,5^ and it allowed us to explore what it is like to be in the “lifeworld” of the participants, considering their personal and social life in the context of temporality, the past, present, and future.

An experienced female qualitative researcher (EEP), who had a background in psychology and prior research experience of injury, conducted the interviews. Interviews explored parents’ experience of injury, treatment, and recovery, and their experience of being asked to include their child in the CRAFFT study. Parents were asked how they felt about the treatments and the CRAFFT study at the time of injury, and at the time of their interview. Semi-structured interviews, with open-ended questions, enabled participants to say what is most important to them, and allowed the researcher to adapt and introduce questions relevant to participants’ accounts of their experience.

Interviews were audio-recorded, transcribed, and managed using NVIVO 12 (QRS Warrington). EEP led the analysis using a reflexive approach to thematic analysis as described by Braun et al.^6^ Analysis was inductive and iterative, with the researcher active in interpreting patterns of meaning in the data. EEP listened to the recordings, read the transcripts, and wrote field notes to gain an understanding of each participant’s world and important elements of their experience. Data were coded line by line, with similar codes grouped to form categories and then into themes. EEP and an experienced qualitative researcher (ET) discussed the data throughout data collection and analysis, and further discussions took place with the co-authors of this article.

Rigour and trustworthiness were achieved by familiarization with the data,^7^ reflection and discussion throughout the analysis, and the inclusion of quotations to illustrate our interpretations. Rich description of the context and methods enables readers to consider the transferability of these findings to other contexts. Data saturation was achieved when no new categories or themes arose from subsequent data collection. The consolidated criteria for reporting qualitative research (COREQ) guidelines informed this article.^8^

### Patient and public involvement and engagement

Parent representatives have been involved in the CRAFFT study and this qualitative study by contributing to: 1) the study design; 2) development of study materials; 3) the CRAFFT study management and steering committees; 4) discussion of the qualitative findings; and 5) the development of this article.

## Results

**Fig. 1:**
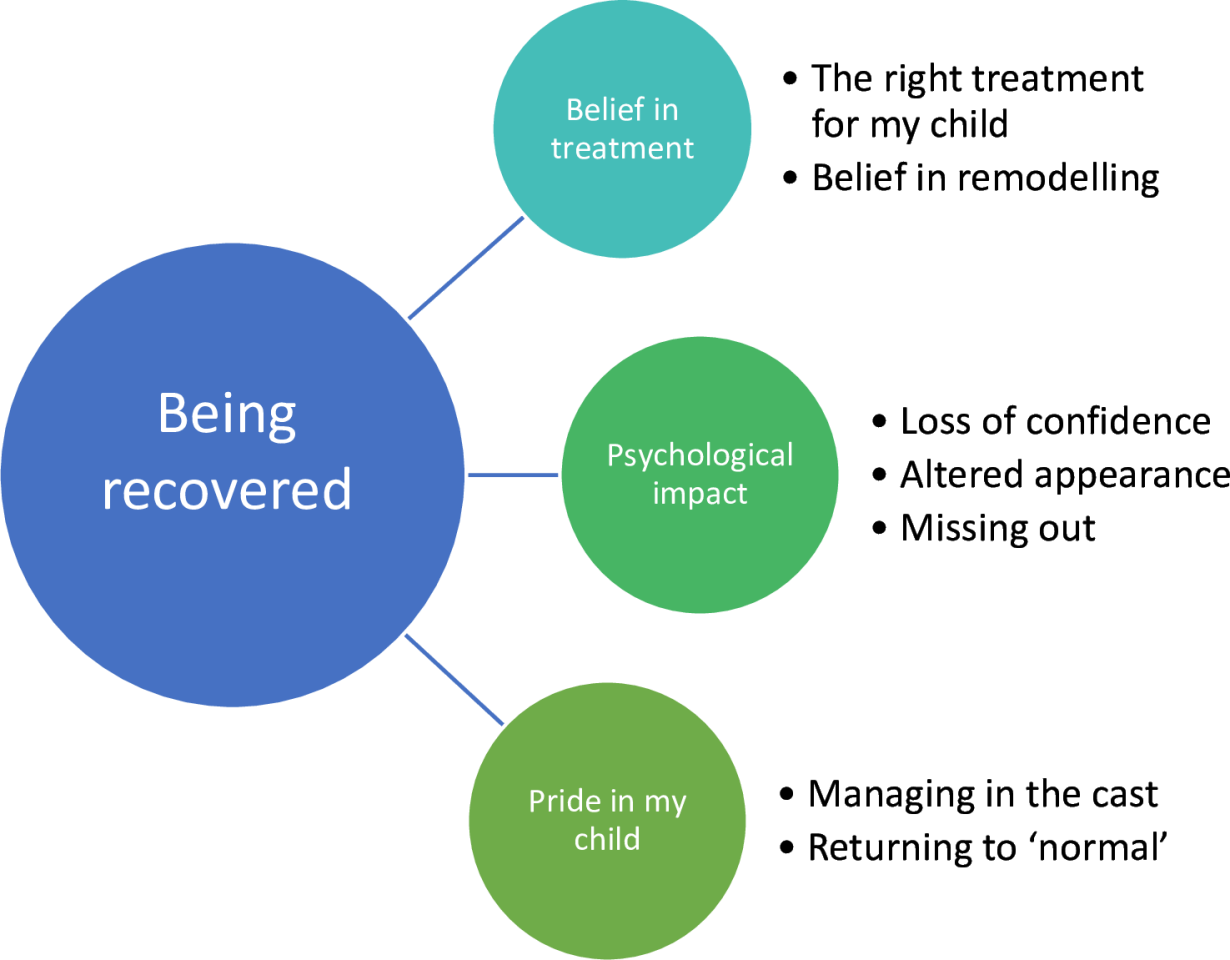
The new theme “Being recovered”, with associated categories.

This study identified the theme “Being recovered” (Figure 1). “Being recovered” conveys the parents’ acceptance and belief in their child’s treatment, their memory of the psychological impact of the injury for their child (despite the passage of time), and their pride in their child’s recovery. Illustrative quotes from participants are presented alongside each category in Tables I and III.

**Table I.** Belief in treatment – illustrative quotes.

Code	Illustrative quotes
The right treatment for my child	“I’m kind of glad he did get the surgery. It sounds like it was quite a bad break because they had to do the open reduction. The surgeon said they rarely had to do that in children. So yes, I just don’t know how it would have gone if he had just got the cast.” (Interview #13, surgical reduction) “Very glad, very glad we participated. I think she’s had a really good result from it, she’s probably recovered quicker in many ways than she would have done with an operation.” (Interview #1, non-surgical casting)
Belief in remodelling	“If she’d had an operation, I might have worried she’d got an infection. When I balance it up logically, I’m very happy that she went down this route. If a friend was in a similar situation and could choose, I would say don’t have the operation, do this because it does work.” (Interview #19, non-surgical casting) “Yes, I’m happy we did it. I mean I’m really happy we did it. We were randomized and I’m really happy we did what we did. I’m happy that we did the trial, I’m happy that we saw in real time how flexible children’s bones are at that age and how they can be remodelled naturally, just by letting them reset.” (Interview #6, non-surgical casting) “Yes, it looks normal, but I think to a medical eye, I think maybe they spotted there was a slight kink or something, it’s very small. If you were like looking at it, as an unknowing person, it looks like a completely normal wrist shape, and yes there doesn’t seem to be anything wonky. They said the small kink in it will straighten out as he gets bigger anyway.” (Interview #11, surgical reduction) “The body can do some pretty amazing stuff, can’t it, that is what’s happening and I’ve seen that it has. When it came out of the cast it wasn’t quite straight and there was a lump there, but it’s straightened out and is reducing. So, what I was told is what I’ve been seeing, so I have no reason really to doubt that. We’ve been really pleased and yes, we’re delighted and feel very lucky actually that the study was going on when this happened to [my child]. I do hope actually that they don’t operate in quite so many cases with children in the future because it seems to have worked out very, very well.” (Interview #9, non-surgical casting) “It’s a lot better than it was. It wasn’t straight when the cast came off, it wasn’t, it was quite bent. The consultant said it would just sort of straighten out as his bone grows. It’s not back to normal but it’s definitely a lot better than it was.” (Interview #13, surgical reduction).

**Table II.** Psychological impact – illustrative quotes.

Code	Illustrative quotes
Loss of confidence	“I mean we’re all super-cautious. I think psychologically, it’s worse than the physical side of it. If it aches, she takes some paracetamol and it’s fine. But psychologically, with this weather [getting worse] she’s very scared of slipping. She’s very cautious when out in public places in case anybody bangs into it. She slipped on the stairs and she dare not move, even look at her arm in case it had broken again.” (Interview #8, non-surgical casting) “I think sometimes he worries when he falls over. He thinks it might break again, but I’ve reassured him that I don’t think it’s any more vulnerable to breaking than a normal arm now.” (Interview #14, non-surgical casting) “I think with him it’s maybe slightly psychological. Sometimes, when he’s playing sport or doing anything he might not give 100%, it’s almost like 90%, because maybe he’s worried about hurting himself again, but physically he can do everything.” (Interview #16, non-surgical casting) “He was a bit apprehensive about doing things like playing football and we didn’t do anything too physical like that. He didn’t do PE (Physical Education) for a couple of weeks, and then he was a bit apprehensive. He fell over once and he was really upset thinking he’d broken it again, but I [said] “No, you’ve just fallen over. Every time you fall over, you’re not going to break it” and it was just about reassuring him. I think it traumatized him a bit that it got broken in the first place, but that’s subsided.” (Interview #15, surgical reduction)
Appearance	“I think she looked quite unhappy [when the cast came off] and said “look at my arm mummy”, but I just said to her “oh, it’s fine”. Then she was quite nervous about moving it, because she didn’t really have a full range of movement straight away. She can do everything with it now and it’s absolutely fine, but it did take a little while.” (Interview #19, non-surgical casting) “He needed to have an operation and I warned him there would be a scar, I warned him there would be a wire sticking out of his arm and it would have to come out. I think he was quite worried about the wire coming out which was out in seconds, but I think he was quite shocked having seen the scar. He never complained when the cast was on, but I think it was almost out of sight, out of mind. When he saw the scar, he almost, he said it was really, really sore but I think it was just seeing it.” (Interview #13, surgical reduction) “It was still quite misshapen, which they told us about, but I don’t think [my child] was quite ready for what that would look like. A few people at school [said] “your arm still looks broken” because it was still quite displaced.” (Interview #12, non-surgical casting) “It’s red and a little bit raised and she doesn’t particularly like it, you can see it and she’s a little bit paranoid about it.” (Interview #2, surgical reduction)
Missing out	“I think the only thing that really frustrated him was the fact that, in and out of school, he couldn’t play with his friends, because we didn’t want him to make it worse. I don’t think he could do sports for 12 weeks and I think that got to him the most, other than that he managed brilliantly.” (Interview #16, non-surgical casting) “I think he just got a bit tired of missing out. It was the snowball effect because it was the summer, he missed sports day and he missed some of his football games and cross-country. He missed out on a few bits and pieces coming towards the end of the school year and stuff.” (Interview #17, surgical reduction)

**Fig. 2:**
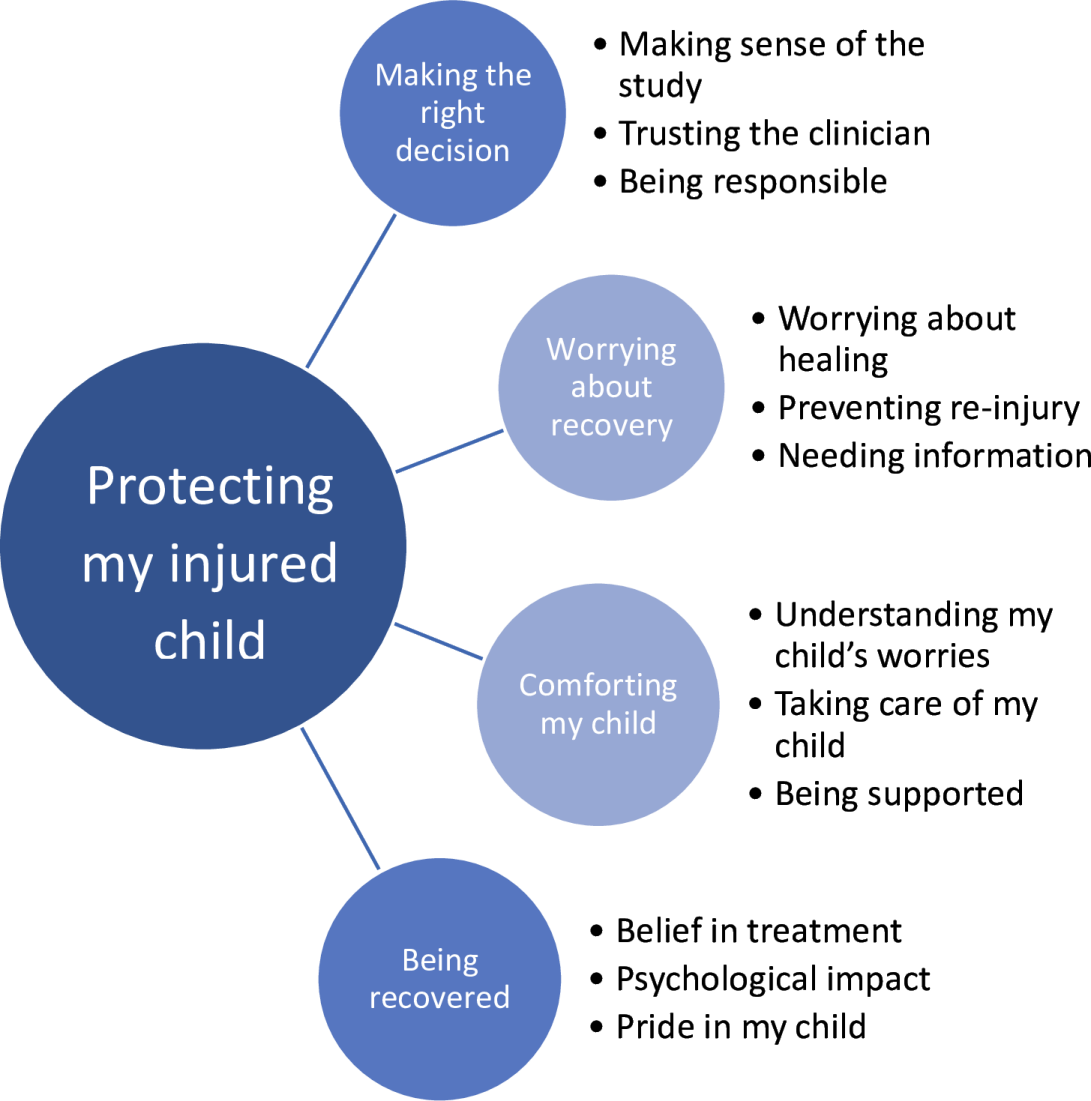
Protecting my injured child throughout recovery: the extended framework.

Three themes were independently identified in this study (interviews five to 11 months post-injury), which were also identified in the interviews we undertook during the early stages of recovery.^2^ These themes were: 1) making the right decision; 2) worrying about recovery; and 3) comforting my child. The findings of the new theme of ‘being recovered’ were combined to provide an extended framework of “protecting my injured child throughout recovery”, (Figure 2). The descriptors of the three themes and categories from our prior study of early recovery, with illustrative quotations from this later study of recovery, are presented in Supplementary Table i.

**Table III.** Pride in my child – illustrative quotes.

Code	Illustrative quotes
Manging in the cast	“I was fully expecting that he was going to be crying and grumpy, and saying it was throbbing or itchy, but he just didn’t really at all. Actually, we were really lucky with the time of year, because it was before the height of summer and it wasn’t really cold. Obviously, we had to sacrifice a few school jumpers where I had to cut the sleeve, but I think you’d have to do that anyway. He was really totally fine and it was probably the best outcome that anyone could ask for really.” (Interview #11, surgical reduction) “It’s awkward showering and managing but she was insistent on going straight back to school as soon as she had the cast. She was actually at school the following day and doing everything for herself. Once we’d heard that she wasn’t going into hospital for an operation she was up and at it.” (Interview #1, non-surgical casting) “He was absolutely fine (with the cast). He didn’t really have any issues with it, yes nothing rubbed or anything like that – he just got on with it, he was very protective of it and if he had it in the sling, he was far more comfortable.” (Interview #17, surgical reduction) “He was amazing. It was on his right hand, and he’s right-handed. We kept him off for a couple of days just to let him get used to the pain relief. When he went back to school, the school were brilliant at helping him with his left hand and letting him use tablets (computers), rather than sort of doing all his work (as normal). In terms of looking after himself, the only thing he couldn’t really do was tie his shoelaces, but he’s not brilliant at that anyway. In terms of washing himself, he made sure to always cover (the cast) in the shower and in the bath. When eating, he managed okay with his left hand and so he did really, really well.” (Interview #16, non-surgical casting)
Returning to normal	“He literally didn’t complain about anything at all after it had happened, literally back to fighting fit. It was like he’d never broken it.” (Interview #15, surgical reduction) “I’ve almost forgotten that he broke it; in a pleasant way. Sometimes he will say he was really pleased with a [football] tackle today and then it sort of reminds me about it. He swims, and the stage that he’s at for a ten-year-old, doing the amount of solid swimming you wouldn’t notice it in the pool at all either, so work-wise and school-wise there is no impact. I’m just amazed how brilliantly he has recovered. We’re really pleased how well he’s recovered, because accidents do happen and can have a long-term impact, but you really would not know it to see him.” (Interview #16, non-surgical casting) “After a couple of weeks, two or three weeks, she was just doing normal stuff with it and she’d do everything normally.” (Interview #19, non-surgical casting)

## Theme – Being recovered

### Belief in treatment

Parents almost invariably believed the treatment their child received was the right treatment for them. Parents of children who had received non-surgical casting expressed amazement that children’s bones can remodel after seeing improvement in the appearance of their child’s wrist.

### The right treatment for their child

Irrespective of their child’s treatment, parents expressed a sense of relief about the treatment that their child received. They felt their child had the right treatment and the best outcome. Parents thought the other treatment may have been suitable for children in different circumstances. They were pleased that their child had not experienced the other treatment, reiterating the risks such as infection and anaesthetic for surgery, and the wrist not straightening for nonoperative treatment.

I don’t think he would have recovered any more quickly if he’d have had an operation. I think there would have been more of a risk, and I think he would have recovered more slowly - so I’m happy that he didn’t have one. (Interview #14, non-surgical casting)

Parents whose child received surgery often expressed relief knowing that their child’s bones were put in the right place. If their child did not have internal fixation with metalwork, parents were glad they did not need it and felt their child had received the best treatment (straight bones without the additional risks associated with fixation). However, when children did have internal fixation, this confirmed to parents that their child’s break was serious and needed surgery.

For me, I personally felt relieved that I had made the right decision (to take part in the trial). I just felt at least I know the bones are in a better place. (Interview #11, surgical reduction)

Parents whose child received non-surgical casting were relieved their child avoided surgery, the scars from wires or plates, and the psychological upset of an operation. Several parents also believed their child’s recovery would have been longer with surgery.

In hindsight, I’m glad she hasn’t had an operation, because she may have had scars on her arm, and obviously there was the whole risk of infection and it slipping. So, I am pleased that she’s not had the operation, but yes just at the time when the cast came off, I was a bit surprised that it didn’t look 100%. (Interview #19, non-surgical casting)

### Belief in remodelling

Parents’ belief in remodelling was evident: they were impressed and amazed to see their child’s wrist straighten. Some parents implied that they did not really believe in remodelling until they saw for themselves. They explained they would now recommend avoiding surgery after a wrist fracture to other parents and they hoped fewer children would have surgery in the future. Parents felt grateful to have had the opportunity to take part in the trial, and for their child to avoid surgery.

I’m happy that we did the trial. I’m happy that we saw in real time how flexible children’s bones are at that age, and how they can be remodelled naturally just by letting them reset. I mean it is just incredible, just to see that happen in real time. So, it has been quite a phenomenon to actually see, wow! Isn’t that amazing that it can actually happen. It’s really amazing to be honest. (Interview #6, non-surgical casting)

Parents had seen great improvement in the appearance of their child’s wrist. Often it looked normal or almost normal, with only a healthcare professional or someone who knew about the injury likely to notice minor imperfections. Parents whose child’s wrist was still not completely straight, regardless of which intervention they received, had a renewed confidence and hope that the wrist would continue to straighten. They had seen improvement, and this had reassured them that their child’s wrist would continue to improve. Others accepted that the wrist might always look as it does now and they were content with that.

It’s not perfect but it’s definitely improved. The last time we went the man said one day you will just wake up, you will look at your arm and you will suddenly realize without you even having been aware of it, that it looks normal again. You can already see, because I looked at it last night in anticipation of your call, and I thought, oh, it really is starting to look a little bit like a normal arm now. (Interview #4, non-surgical casting)

## Psychological impact of injury

Parents reflected upon the psychological impact of injury for their child, which was often more difficult than the physical recovery. A broken wrist was traumatic for children who often experienced a loss of confidence, worry about the appearance of their wrist, and were upset to miss out on activities while recovering.

### Loss of confidence

Parents described their child’s loss of confidence after injury. After the cast was removed, some children were nervous of their arm being exposed without protection and a visual reminder to others to be careful. They were worried that they would break their arm again and were upset if they fell over. Some children were nervous to resume activities, play outside, or walk outside if the ground was wet after rainfall. However, for most children, the loss of confidence and fear of re-injury subsided with time, and they learnt to trust their arm again.

She hated the fact that it was coming off. She was terrified because that protective layer had gone and so it probably took her a couple of weeks to feel a bit okay, safe and secure. (Interview #8, non-surgical casting)

### Appearance

Some children were upset by the appearance of their arm, commenting on the look to their parents. They were conscious that their wrist looked wonky or disliked the scars from Kirschner (K)-wires. A minority of children faced comments from others, such as sports leaders or school friends, and needed to explain to others that their wrist was all right to use, despite still looking broken.

I mean in that first few weeks I think with dancing it was the most noticeable because she was in a leotard. So, her arms were bare and she did sometimes say to me “oh my arm does look a bit wonky still doesn’t it mummy” and I’d say it’s fine, it’s still sorting itself out. She did notice that a little bit, but as I say, now it’s totally okay. (Interview #19, non-surgical casting)

I think the only thing that she probably struggles with, at the moment, is the scar. Yes, she just said “look at my scar, do you think it will fade?” and I said “yes, it will fade”. It’s a bit red at the moment because it’s recent, but it will fade. That’s the thing that probably affects her more. (Interview #2, surgical reduction)

### Missing out

Children had experienced frustration when restricted by their injury, and this was often what upset them most during their recovery. Both treatment groups missed activities, such as swimming, sports, and playing outside with their friends. Some parents increased the recovery time, beyond that suggested by clinicians, to ensure their child’s wrist had healed before restarting activities. However, some parents found out their child had re-started activities, such as playing football or doing cartwheels before they were allowed. A minority of children received a waterproof cover for their cast to allow them to continue to swim and shower.

She was so frustrated and was so upset when I said “no, no, we need to wait a bit longer”. I did wait a lot longer than what they said, just to make sure. Now, she’s doing everything that she did before. (Interview #2, surgical reduction)

## Pride in my child

Parents were proud of their child and how they coped after their injury. Parents described their child as brave, composed, and resilient, coping with the hospital environment despite being in pain and being afraid of potentially having an operation.

### Managing in the cast

Parents were impressed with how well their child coped while their arm was in a cast. Children often adapted quickly and found ways to go about their daily activities despite being restricted. They managed tasks like dressing, washing, eating, and doing schoolwork, and were careful to prevent their cast from getting wet. Children were often keen to get back to school and resume normal activities, as much as they were able. Parents expected their child to complain and struggle, and were proud and surprised by their resilience. However, a minority struggled with the recovery affecting their schoolwork and motivation.

She was very, very resilient within a very short space of time. She figured out how to put her belt on in the car without needing any help and there was very little she couldn’t do after a very short space of time. (Interview #12, non-surgical casting)

It sounds trivial but she couldn’t hold a pen properly and that’s quite important to a child at school. Her handwriting was messy, it made her not want to work and she had to use a laptop. (Interview #8, non-surgical casting)

### Returning to normal

At the time of interview, for the majority of families, the injured wrist was almost forgotten and in the past. Children had all returned to their prior activities, including sports such as dancing, gymnastics, and football. For most children, recovery was gradual, gaining confidence as they learnt to trust their wrist again. A minority of children experienced little fear and eagerly returned to their sport.

He really just didn’t stop. He tried to carry on doing everything that he was doing before and he didn’t really let anything stop him. If anything, he felt that he was a bit unbreakable! (Interview #6, non-surgical casting)

## Discussion

This study identified the theme “Being recovered”, which comprised three categories: 1) belief in treatment where parents are content that their child received the right treatment for them; 2) psychological impact where parents remember their child’s loss of confidence, worry about the appearance of their wrist, and upset at missing out as a consequence of their injury; and 3) pride in their child where parents are impressed with how well their child coped while recovering. This theme extends our original framework “protecting my injured child’ identified in the first three months of recovery”^2^ to become “protecting my injured child throughout recovery”.

The themes in this revised framework highlight three key elements: 1) parents accepted the non-surgical casting as a treatment; 2) parents and (from the parents’ perspective) children had differing experiences, but both had generic concerns about recovery – these were similar across the two treatments, but there were also specific concerns dependent upon treatment; and 3) families required further educational support during early recovery.

Our findings demonstrate that by one year after injury, parents were content with their child’s treatment irrespective of the intervention arm (i.e. surgical reduction or non-surgical casting), believing they had received the best treatment for them, as found in other studies of injury.^9,10^ Up to three months post-injury, some parents had experienced doubt about their child’s treatment or regret about their decision to take part in the CRAFFT study,^2^ but this study indicates that these feelings dissipate as children recover. In the non-surgical casting group, parents expressed relief and renewed belief in remodelling. They were glad that their child had avoided surgery and impressed by the continued improvement in appearance of their child’s wrist. In the surgical reduction group, parents felt their child’s wrist was “fixed” and also believed the appearance of their child’s wrist, in particular scars, would continue to improve with time.

Parents reported that they and their children had physical, psychosocial, and emotional concerns throughout recovery. Key challenges for parents and children were shared across the treatment groups (as shown in Table IV), with additional specific concerns dependent upon treatment.

**Table IV.** Key challenges for recovery.

**Key challenges for children**	**Key challenges for parents**
Fear of re-injury	Insufficient knowledge about recovery
Returning to activities	Uncertainty about care once their child’s cast was removed
Adapting and managing in the cast	Overcoming negative emotions such as: worry about healing, re-injury, the appearance of their child’s wrist, and regret or doubt about their treatment decision
Overcoming a loss of confidence	
Upset at the appearance of their wrist	

Certain elements of recovery were particularly challenging. Once the cast was removed, parents felt there was a loss of protection, they felt unprepared, and needed information about what to expect and what their child should or should not do with their wrist. Children frequently experienced a loss of confidence and found the appearance of their wrist upsetting. Some were upset that their arm looked wonky, while others were upset by the scars from K-wires.

**Fig. 3:**
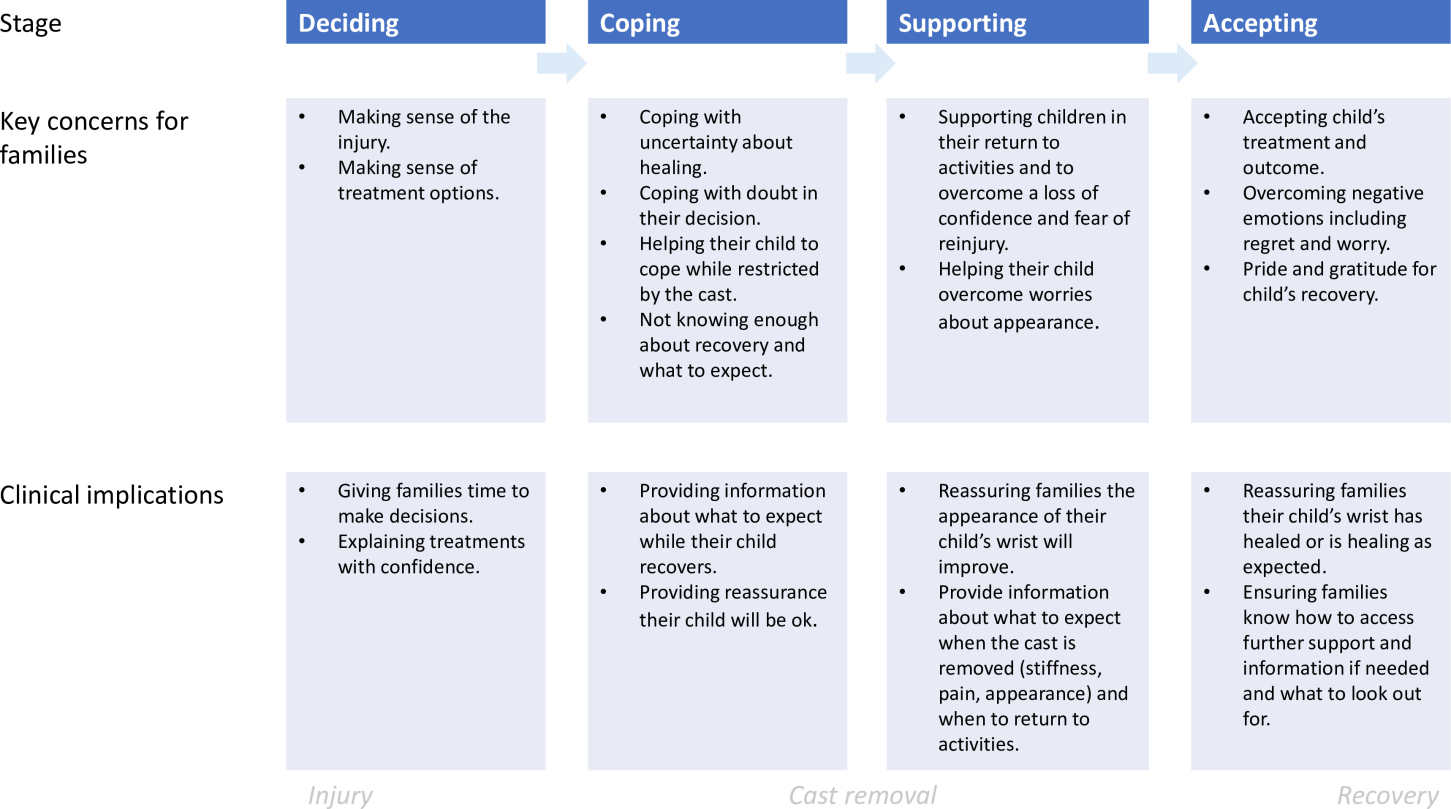
Key challenges and implications for practice during recovery.

Our study suggests that we need to explore the best way to support families and help them to overcome the negative emotions arising from the uncertainty of their child’s recovery, such as worry about healing, doubt, and regret in their decision, particularly in the early stages of recovery. The value of providing psychosocial support to injured children and their families is acknowledged by clinicians.^11^ They can help families by addressing their concerns and providing reassurance that the child’s wrist will heal, and that the appearance will improve with time.^2^ Feeling supported can help parents to cope with anxiety of having an injured child.^12^ However, resources necessary for clinicians to help families may be lacking due to: 1) pressured everyday clinical situations; 2) the time, space, and skills to help parents process emotions; and 3) surgical knowledge, expertise, and confidence to explain remodelling to parents.^2,11,13^ Greater educational support for families, which provides clear information about what to expect when the cast is removed, and how and when to return to usual activities to alleviate concerns about re-injury, would enable parents and their child to cope with the experience of uncertainty throughout recovery (Figure 3).

This study used a purposeful sample of mothers and fathers from across the UK. Our sample included parents of children who received both study interventions, and one parent who declined participation in the CRAFFT study. This participant was included because parents who declined participation may have different views, concerns, and experiences to those who took part in the CRAFFT study. The findings of this study strengthen our framework “protecting my injured child” (recovery up to three months).^2^ This independent study (recovery up to 11 months) found three of the themes identified within our prior study. Further research with children would provide additional insight into the experience of recovery from injury and what is important to children.

By one year after injury, parents in both treatment groups considered their child “recovered”. They had overcome early concerns about healing, including specific concerns in the non-surgical casting group about remodelling, the appearance of the wrist, and coping after cast removal. Greater educational support for families during the early stages of recovery would enable parents and their child cope with the uncertainty of recovery, particularly addressing the loss of confidence, worry about re-injury, and the appearance of their wrist when the cast is removed.

**Take home message**

- By one year after a displaced distal radius fracture, parents considered their child “recovered”. They had overcome early concerns about healing, including specific concerns about remodelling for those treated with non-surgical casting, the appearance of the wrist, and coping after cast removal.

- Greater educational support for families during the early stages of recovery would enable parents and their child to cope with the uncertainty of recovery, particularly addressing the loss of confidence, worry about reinjury, and the appearance of their wrist when the cast is removed.

## Data Availability

The data that support the findings for this study are available to other researchers from the corresponding author upon reasonable request.
